# Eustachian Tube and Otoscope

**Published:** 1864-10

**Authors:** 


*Eustachian Tube and Otoscope.*—Mr. Toynbee says that by demonstrations with Politzers Otoscope, the Eustachian Tube is only open during deglutition. He instances the fact that air can be forcibly injected into the tympanum by holding the nose and blowing with the mouth shut. This air can only be got rid of by deglutition, which opens the tube and lets it out.

